# Case report: An evaluation of early motor skills in an infant later diagnosed with autism

**DOI:** 10.3389/fpsyt.2023.1205532

**Published:** 2023-06-19

**Authors:** Lauren G. Malachowski, Margaret-Anne Huntley, Amy Work Needham

**Affiliations:** Department of Psychology and Human Development, Vanderbilt University, Nashville, TN, United States

**Keywords:** autism, infancy, fine motor skills, visual attention, emulation

## Abstract

Researchers and clinicians are increasingly interested in understanding the etiology of autism spectrum disorder (ASD) and identifying behaviors that can provide opportunities for earlier detection and therefore earlier onset of intervention activities. One promising avenue of research lies in the early development of motor skills. The present study compares the motor and object exploration behaviors of an infant later diagnosed with ASD (T.I.) with the same skills in a control infant (C.I.). There were notable difference in fine motor skills by just 3 months of age, one of the earliest fine motor differences reported in the literature. In line with previous findings, T.I. and C.I. demonstrated different patterns of visual attention as early as 2.5 months of age. At later visits to the lab, T.I. engaged in unique problem-solving behaviors not demonstrated by the experimenter (i.e., emulation). Overall, findings suggest that infants later diagnosed with ASD may show differences in fine motor skills and visual attention to objects from the first months of life.

## Introduction

Autism spectrum disorder (ASD) is a neurodevelopmental disorder characterized by persistent social and behavioral differences (1). Children with autism spectrum disorder (ASD) typically do not receive formal diagnoses until “core features” (i.e., social communication differences) emerge in toddlerhood (1, 2). However, detection and intervention *before* the onset of these behavioral differences may be especially beneficial for understanding etiological mechanisms and optimizing child outcomes (1–3). Early developments in the motor domain may offer important insight into the etiology of ASD. Given close ties between social skills and motor skills, as well as rapid changes in motor skills during infancy, some researchers have proposed that the earliest signs of ASD are likely to be motor-related (4–6).

In infancy, motor skills like reaching, grasping, and crawling facilitate object exploration, a primary means through which infants collect sensory information and build knowledge about the world (7). From a developmental cascades perspective, these early interactions with objects, and the diverse learning opportunities they provide, accumulate across time to drive developmental change (8–10). In fact, object exploration has been found to predict later cognitive, language, and even social development (11–13). Thus, examining object exploration and associated motor skills in infancy may be particularly useful in the search for early markers of ASD (14).

In line with this developmental cascades framework, recent work has documented differences in motor skills (1, 6, 15–17) and object exploration behaviors (18–20). However, many of these studies focus on mid- to late- infancy (6+ months), and more work is needed to understand emerging differences in the first months of life. Additionally, many previous studies recruit infant samples at elevated likelihood for developing ASD, which may introduce confounding factors that obscure findings (i.e., characteristics of infants at higher likelihood for ASD that are not directly relevant to ASD). Retrospective analyses of infants with confirmed ASD diagnoses can help to eliminate this concern.

The present study is a longitudinal, retrospective case study comparing the exploration and motor behaviors of two infants– one later diagnosed with ASD and one age-matched control– between 2.5 and 24 months of age. Both T.I. and C.I. were male, White, and non-Hispanic. Both of their mothers had graduate degrees. Both infants were born full-term and approximately the same age at each visit. To the authors' knowledge, this study is the first retrospective case study of its kind. The primary aim was to identify potential early motor and object exploration markers to guide future studies on the etiology and development of ASD.

## Methods

Diagnosis information was obtained via a brief survey sent to previous participants in an infant research lab. The survey asked parents to report on any diagnoses their child may have received since their last lab visit. One family reported that their child (“T.I.” for “target infant”) had been diagnosed with ASD and a speech delay. T.I. had previously participated in a longitudinal study in the lab. The original longitudinal study was designed to assess basic developmental processes and did not involve recruitment of infants at elevated likelihood of developmental disorders. A participant from the same longitudinal study was selected as a sex- and age-matched control (“C.I.” for “control infant”). C.I.'s parents confirmed the absence of any developmental delays or diagnoses by 3 years of age.

According to T.I.'s mother, T.I. was diagnosed with ASD and speech delay at 19 months of age via the Vineland Adaptive Behavior Scales (21); the Mullen Scales of Early Learning (22); and the Autism Diagnostic Observation (23). The family had the ASD diagnosis confirmed by the state's early intervention system. T.I. had no family history of ASD. When the mother endorsed T.I.'s diagnosis in the survey, T.I. had already begun speech therapy, occupational therapy, physical therapy, and developmental therapy.

**Figure 1:**
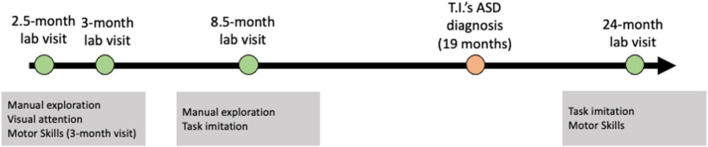
Timeline of lab visits and assessments.

As a part of the original study, T.I. and C.I. visited the lab four times at the following ages: 2.5, 3, 8.5, and 24 months (this last visit was post-diagnosis for T.I., and lab members were first made aware of the diagnosis during this visit). See Figure 1 for a visual timeline. Each laboratory session consisted of structured play sessions with the infant sitting on a caregiver's lap at a semicircular table. At the 2.5- and 3-month visits, various age-appropriate objects were placed on the table within reach of the infant for 30- or 60- s intervals. At the 8.5- and 24-month visits, infants were asked to imitate multiple object-related actions, such as building a tower with blocks. All laboratory sessions were filmed with a 4-way video camera system for future coding. Additionally, the Early Motor Questionnaire (EMQ), a parent-report questionnaire assessing children's early motor skills in the context of everyday situations, was administered at 3 and 24 months (24). The EMQ was not administered at every visit due to the close temporal proximity of visits as well as efforts to reduce participant burden. The EMQ assesses gross motor skills (49 items), fine motor skills (48 items), and perception-action skills (31 items). The EMQ has demonstrated concurrent and predictive validity when compared to standardized assessments (e.g., Mullen Scales of Early Learning; 21).

Quantitative and qualitative analyses were conducted to compare T.I. and C.I. on motor and object exploration behaviors. Quantitative analyses of video footage were conducted by coders blind to diagnosis information. A detailed coding scheme was developed based on previous work (13). Using Datavyu video coding software (25), coders marked the onsets and offsets of pre-determined behaviors (looking, touching, mouthing, and rhythmic play). Each behavior was double-coded, and final codes were determined after coders discussed and resolved any discrepancies. Total durations of each behavior were then calculated for each infant and each visit. Quantitative analyses also included scores from the Early Motor Questionnaire completed at 3 and 24 months. T.I.'s scores in the gross motor and fine motor domains were calculated and compared to the mean scores for the entire study sample. The original study sample was comprised of 49 infants (56% female). The racial breakdown of the sample was: 84% White, 8% Asian, 6% Black, and 2% Pacific Islander. This was a highly-educated sample, with 53% of mothers having earned a graduate degree.

Qualitative analyses were conducted by the second author, who was unblinded to diagnosis information. These analyses were conducted by carefully examining the video footage of T.I. and C.I. side-by-side and taking detailed notes regarding observable differences in behavior. These qualitative analyses offer insights into behaviors not specified in the video coding schemes.

## Results

### Parent-reported gross and fine motor skills

Table 1 displays a comparison of scores on the EMQ between T.I., C.I., and the overall sample mean. T.I.'s gross motor score was similar to both C.I.'s score and the sample mean at the 3-month visit but substantially lower by the 24-month visit. Most notably, T.I.'s 3 month fine motor score (0) was more than 3 standard deviations below the sample mean (M = 0.67, SD = 0.20). A score of 0 indicates that the infant has not yet demonstrated any of the fine motor behaviors listed on the questionnaire. Examples of fine motor behaviors that other 3-month-old infants demonstrated were: “opens the fingers of each hand spontaneously,” “brings hands together near the face, chest, or tummy,” and “tightly holds onto a toy placed into his/her hand” (24). T.I.'s mother verbally noted at the 2.5-month visit that T.I. liked to tuck his thumb into the palm of his hand. The experimenter noted on a study documentation form that she had trouble opening T.I.'s fingers to place a rattle in his hand.

**Table 1:** EMQ score comparison.

	**T.I**.	**C.I**.	**Full sample**
Gross motor 3 months	0.65	0.67	0.71 (0.18)
Fine motor 3 months	0	0.85	0.67 (0.20)
Perception-action 3 months	1.26	1.48	1.21 (0.27)
Gross Motor 24 Months	3.37	3.71	3.80 (0.15)
Fine motor 24 months	2.60	3.33	3.13 (0.31)
Perception-action 24 months	3.29	3.81	3.77 (0.21)

Means are presented with standard deviations in parentheses. The full sample statistics include both T.I. and C.I.'s scores.

### Manual object engagement

**Figure 2:**
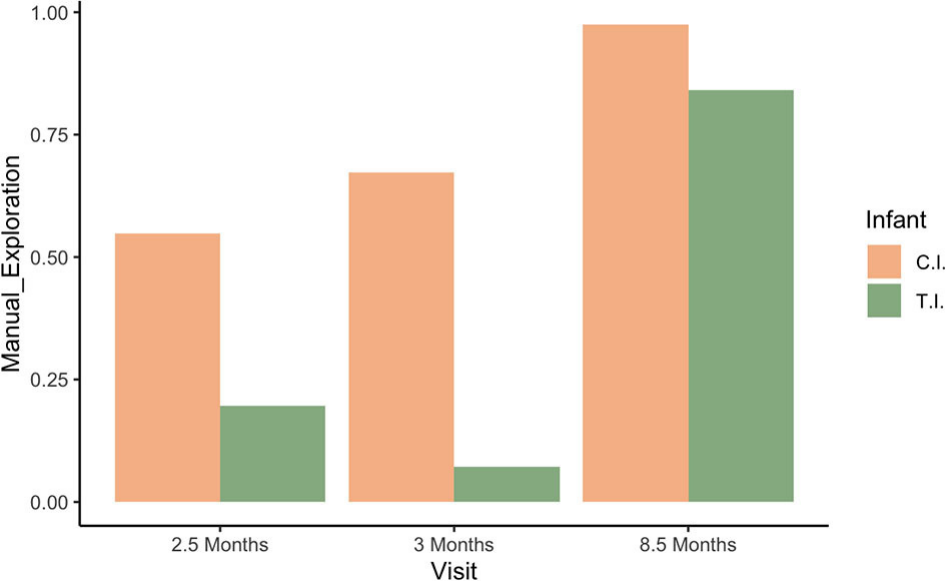
Manual object exploration by visit and infant.

Results from video coding (quantitative analyses) revealed that T.I. spent less time manually engaging with presented objects than C.I. at the first three visits (see Figure 2).

**Figure 3:**
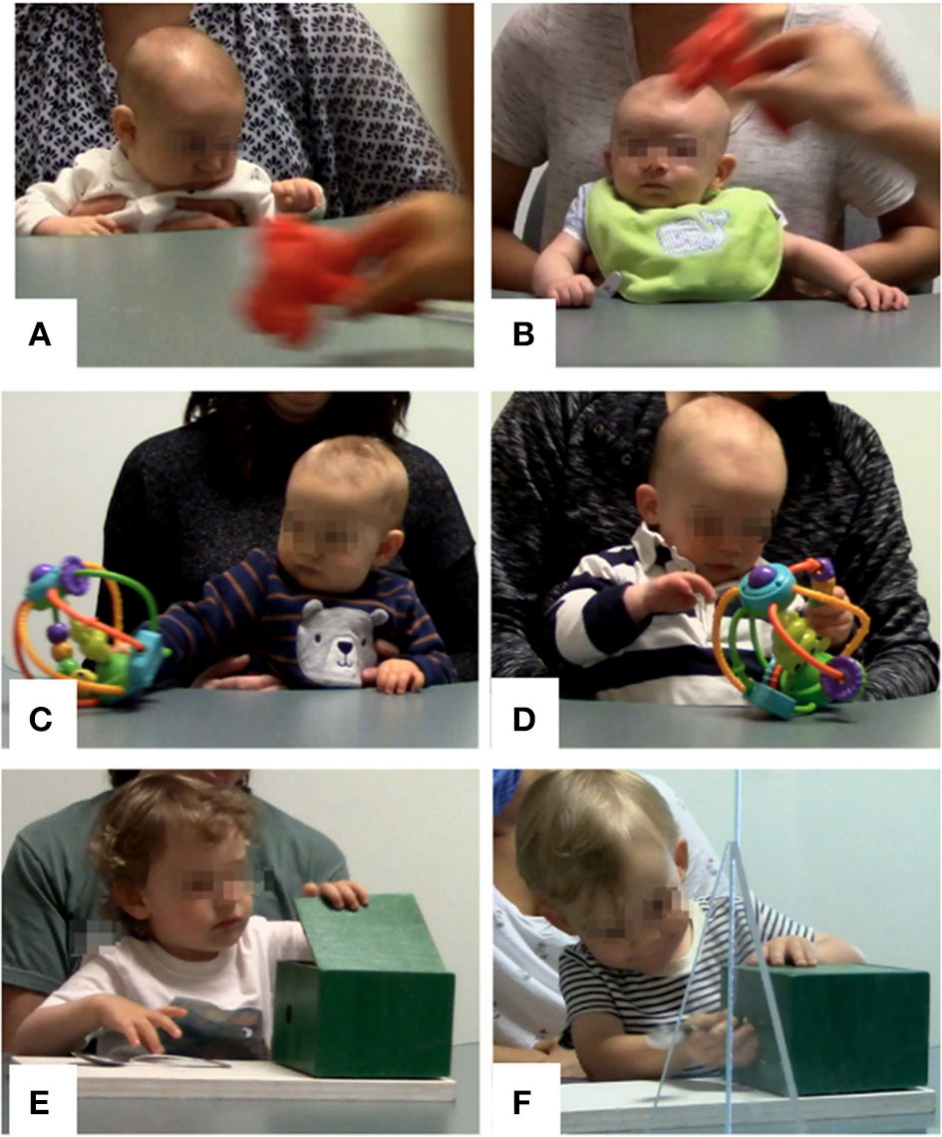
**(A–F)** Images of T.I. and C.I. in the lab.

At the 8.5-month visit, T.I. engaged in more rhythmic play than C.I. (e.g., repeatedly sliding the activity ball across the table; see Figure 3C). T.I.'s mother noted during the filmed session that T.I. liked to slide his hands and toys across the table at home. In contrast, the **second** author noted that C.I. spent more time manipulating the individual components of the activity ball (see Figure 3D).

### Visual attention

At 2.5 and 3 months, T.I. demonstrated a longer latency to visually attend to presented objects compared to C.I., as measured by the number of seconds that passed between the presentation of an object and the onset of the first “look” code. When the experimenter attempted to direct T.I.'s attention to an object, T.I. often took up to 10 s to visually orient. C.I. did not demonstrate this same behavior and tended to orient quickly to new objects (Figure 3B). The second author noted that T.I. displayed a strong preference for looking down at his hands and at the table's surface (Figure 3A). Compared to C.I., T.I. rarely shifted his attention–looking at his hands, the toy, and the table surface continuously for up to 60 s. At the 8.5-month visit, T.I. no longer looked at his hands, but similarly maintained visual attention to presented objects for periods of up to 60 s. In contrast, C.I. frequently shifted his attention between the toy, his mother, and the experimenter.

### Task imitation

At the 8.5-month visit, the experimenter stacked a set of 5 blocks to build a tower and asked the infant to imitate this behavior. Neither infant successfully stacked any blocks. As with the activity ball, T.I. slid the blocks back and forth across the table. The same blocks task was repeated at the 24-month visit. Both C.I. and T.I. successfully built a block tower. C.I. proceeded to knock down the tower, just as the experimenter had demonstrated. In contrast, T.I. accomplished the same goal by removing each block from the tower one at a time to create a straight line of blocks on the table.

A second imitation task at the 24-month visit involved inserting a metal spoon into the side of a lightbox to activate a display of lights. C.I. inserted the spoon into a hole in the side of the lightbox, just as the experimenter demonstrated (see Figure 3F). In contrast, T.I. explored the box itself and discovered that the top of the box could be removed to reveal the lights inside (see Figure 3E). No other child in the study noticed the removable top, which was designed to be discreet (i.e., all painted the same color). T.I. then used his finger, instead of the tool, to activate the light, a solution that was not modeled by the experimenter.

## Discussion

Analyses reveal behavioral differences between T.I. and C.I. in four domains: fine motor skill, manual object exploration, visual orientation, and task-imitation. First, T.I.'s parent-reported fine motor skills at both 3 and 24 months were substantially lower than C.I.'s. This aligns with previous work reporting less advanced fine motor skills in 6-month-old infants with a higher familial likelihood for developing ASD (26), perhaps due to atypical organization of the primary motor cortex (4, 27). However, the present study supports and extends these findings by suggesting that these differences in fine motor skill may be detectable as early as 3 months of age. If this finding is replicated in future studies, very young infants' tendencies to open their fingers, grasp objects, and bring their hands to midline should be further investigated as predictors of ASD. If found to reliably predict symptoms of ASD, fine motor items from the Early Motor Questionnaire may be promising targets for early ASD screening.

Second, consistent with previous literature (18), T.I. spent less time than C.I. looking at (28–32) and manually exploring (18) presented objects. The qualitative findings expand upon existing work by identifying more specific potential markers of ASD; namely, visual attention to the hands and nearby surfaces. Because visual attention and learning are closely related, early differences in visual attention behaviors may accumulate to impact learning (1).

Lastly, at 24 months, T.I. reproduced task goals but did not directly imitate the experimenter's actions with blocks and tools. These findings support previous work suggesting that children with ASD tend to engage in less direct imitation than their typically-developing peers (33–35). Instead, children with ASD are more likely to emulate, or reproduce a goal using methods not observed (35).

These findings must be considered in light of the natural limitations of a case study. ASD can present in many different ways. Additionally, infants' individual differences (e.g., temperament) and experiences with objects may shape their object-related behaviors produced in the lab setting. T.I. and C.I. in the present study may have differed on a number of non-ASD related attributes. Thus, replications of the present study's findings are needed before direct application in clinical settings. Additionally, given sex-related differences in ASD presentation and in general motor development, it will be important to assess early motor signs of ASD in female infants as well.

## Conclusion

Overall, the present study's findings build on previous literature by identifying potential early-emerging signs of ASD in the motor and object exploration domain. Key findings include differences between T.I. and C.I. in parent-reported fine motor skill by 3 months of age, differences in manual and visual engagement with objects, and differences in task-imitation. Future work should explore fine motor skills (i.e., spontaneous finger movement) as possible indicators of ASD in early infancy.

## Data availability statement

The original contributions presented in the study are included in the article/supplementary material, further inquiries can be directed to the corresponding author.

## Ethics statement

Ethical review and approval was not required for the study on human participants in accordance with the local legislation and institutional requirements. Written informed consent to participate in this study was provided by the participants' legal guardian/next of kin. Written informed consent was obtained from the individual(s), and minor(s)' legal guardian/next of kin, for the publication of any potentially identifiable images or data included in this article.

## Author contributions

LM and AN contributed to the conception and design of this study. LM conducted quantitative analyses. M-AH conducted all qualitative analyses of videos. All authors contributed to the article and approved the submitted version.
